# Firefighters’ Expertise in Locating Sounds

**DOI:** 10.3390/brainsci16050492

**Published:** 2026-04-30

**Authors:** Isabel Tissieres, Stephanie Clarke, Sonia Crottaz-Herbette

**Affiliations:** 1 Service Universitaire de Neuroréhabilitation, CHUV|Centre Hospitalier Universitaire Vaudois, Faculté de Biologie et de Médecine, Université de Lausanne, 1011 Lausanne, Switzerland; isabel.tissieres@crr-suva.ch (I.T.); sonia.crottaz-herbette@chuv.ch (S.C.-H.); 2 Service de Psychothérapie, Clinique Romande de la Réadaptation, SUVA, 1950 Sion, Switzerland

**Keywords:** auditory expertise, sound localisation, spatial cues, auditory space, firefighters, sound object segregation

## Abstract

Background: Firefighters search for survivors in low-visibility environments and have documented expertise in spatial orientation and wayfinding. No studies so far have investigated their ability to make use of auditory spatial cues. Methods: The performance of 41 professional firefighters and 24 control subjects to localise sounds played in a dark environment at 9 azimuthal positions was assessed using (i) meaningless single sounds and (ii) meaningful sounds presented with two simultaneous distractors. Localisation errors were analysed with a 3-way ANOVA Task × Position × Group. Results: The analysis yielded significant main effects of Task, Position and Group. The localisation errors tended to be smaller when localising meaningless single sounds (main effect Task), for items presented at central as compared to peripheral positions (main effect Position), and by firefighters rather than controls (main effect Group). The interactions Position × Group and Task × Position were significant; firefighters performed more accurately at peripheral positions than controls, and meaningless single sounds yielded fewer localisation errors at the periphery than meaningful sounds presented with distractors. Conclusions: Professional firefighters perform better than controls at explicit localisation of sounds, including in conditions where sound objects need to be segregated from distractors by spatial cues. These results suggest that firefighters have acquired expertise in the explicit and implicit use of auditory spatial cues. The proficiency of firefighters needs to be taken into consideration when planning and carrying out interventions.

## 1. Introduction

During interventions, firefighters search for survivors in low-visibility environments, having undergone specific training to that effect. Several studies have reported that they perform well at wayfinding [1] when they can rely on spatial knowledge [2] or when they are not hindered by fear of confinement [3]. Since the search for survivors in a dark environment is likely to be facilitated by auditory spatial information, professional firefighters may excel at localising sounds, including in noisy environments. No studies so far have examined this aspect. Two distinct aptitudes would need to be considered [4]. One involves the localisation of sounds explicitly by word or by deed [5,6]. The other involves the use of auditory spatial cues for the segregation of simultaneously occurring sound sources [7,8].

Two lines of evidence indicate that auditory spatial processing is malleable. First, overt localisation of sound sources is improved by training. It is typically tested with tasks of discrimination or identification of sound locations in free-field condition, simulated by interaural time or intensity differences, or modelled by head-related transfer functions [5,6]. Psychophysical investigations have demonstrated that positions of pure tones, simulated by interaural time or intensity differences, were better discriminated after a nine-day training period [9]. The improvement was shown to continue progressively throughout a three-week training period [10]. The effect of a brief intervention was investigated behaviourally and with auditory evoked potentials [11]. A brief 40 min training was shown to lead to more accurate spatial discrimination of white noise bursts, which were lateralized by interaural time differences. Following this brief training, the improvement in performance was short-lived (<6 h) and did not generalise to untrained positions. This brief intervention modulated the cortical representations of the trained sound positions. Second, subjects adapt to changes in auditory cues, which are relevant for sound localisation. The shape of the outer ear determines the perception of spectral cues and impacts the sound localisation along the vertical axis [12,13]. Several studies have investigated how subjects adapt to altered shapes of their outer ears. Immediately after moulds were applied, sound localisation performance was shown to deteriorate before improving progressively over the next days [14]. When the moulds were removed 6 weeks later, localisation without moulds was found to be as accurate as before the shape alteration. The adaptation to novel spectral cues was reported to be faster when sensorimotor training and augmented feedback were used [15].

Due to their long-term exposure and specific training, firefighters may have acquired expertise in the localisation of sounds, helping them to find where the person to rescue is, for example. In addition, they have most likely acquired expertise in segregating relevant sounds in a noisy environment. We have investigated the auditory spatial abilities of professional firefighters and compared them to those of control subjects. We postulated that professional firefighters are likely to be more proficient at explicit localisation of sounds, even in conditions where auditory spatial cues need to be used implicitly to segregate simultaneous sounds.

Two specific hypotheses were tested:

(i) Professional firefighters perform better than control subjects at localising meaningless sounds that are presented individually in a dark environment.
(ii) Professional firefighters perform better than control subjects at localising meaningful target sounds that are presented in a dark environment at the same time as distractor sounds.

Whereas the first hypothesis focuses on explicit sound localisation, the second hypothesis tests the interplay between explicit and implicit use of auditory spatial cues.

## 2. Methods

### 2.1. Participants

Sixty-five participants were included in this study: 41 professional firefighters and 24 control subjects. Professional firefighters were aged 24–54 years (mean ± SD: 37.6 y ± 7.6 years); all were male, and six were left-handed. Their professional activity and training amounted to 12.4 ± 7.3 years. None of the behavioural measures correlated significantly with the training duration (as assessed with Pearson correlations and Bonferroni correction for multiple comparisons). The control subjects were aged 23–45 years (mean ± SD: 33.9 y ± 6.6 years); 15 were male and 2 left-handed. None of the participants had a history of psychiatric or neurological disorders, all reported normal or corrected-to-normal vision and for all audiometry was within normal limits, including detection thresholds which did not differ by ≥15 dB between the ears. The study has been approved by the Ethics Committee of the Canton de Vaud, Switzerland, and all participants provided written informed consent according to the procedure.

### 2.2. Experimental Design

Each participant performed a block of auditory localisation tasks and another block of visual and attentional tasks. The order of blocks was counterbalanced between the subjects of each group.

### 2.3. Auditory–Spatial Tasks

**Figure 1:**
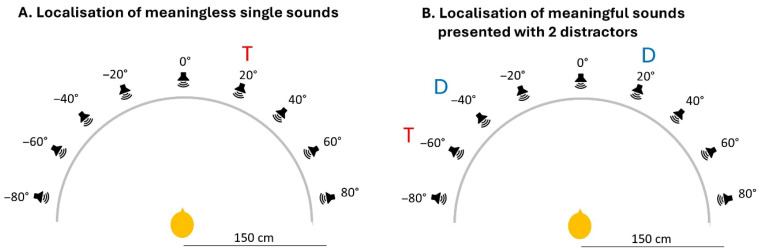
Experimental set-up used in sound localisation tasks and arrangement during exemplar trials. Speakers were spaced 20 degrees apart along a semicircle, at a distance of 1.5 m from the participant, at azimuthal positions of −80°, 60°, 40°, 20°, 0°, 20°, 40°, 60° and 80°. An acoustically transparent curtain, represented here by a grey line, concealed the speakers from the participant’s view. (**A**) Localisation of meaningless single sounds. Target sound (T): a noise burst of 500 ms duration was presented at one of the azimuthal positions (here et 20°). (**B**) Localisation of meaningful target (T) sounds presented with two simultaneous distractors (D). Target was a sound produced by humans, here a woman sneezing, at one position (here −60°), together with two distractors, here a helicopter flying at −40° and glass breaking at 20°.

Sound localisation tasks were performed in a dark, soundproof room. Nine loudspeakers were arranged in a half-circle at 1.5 m from the subject and at the relative positions of −80° (far left), −60°, −40°, −20°, 0° (straight ahead), 20°, 40°, 60° and 80° (far right; Figure 1). A black, acoustically transparent curtain concealed the loudspeakers from participants’ view. Participants indicated the position of target sounds with a laser beam, which was emitted by a laser robot. Between trials the laser position was at 0°, and participants were asked to fixate it. After hearing the sound, the subjects directed the beam to the perceived position of the sound by manipulating a joystick with their dominant hand. They pressed a button when the laser beam reached the perceived position. Before testing, each participant was instructed in how to use the pointing device and performed 10 practice trials with meaningless single sounds. The tasks were programmed using Tucker Davis Technologies (Alachua, FL, USA).

Two tasks were used:

(i) Localisation of meaningless, single sounds, taken from a battery of 8 different broadband noise samples, all 500 ms in duration, including 10 ms linear rise and fall time. Throughout the trial, sounds were presented in a pseudo-randomised fashion 8 times at each of the 9 positions. The task lasted 6 min and 30 s.
(ii) Localisation of meaningful target sounds, which were presented with two simultaneous distractors. Targets were sounds produced by humans (baby crying, woman screaming, man coughing, woman sneezing, man and woman laughing, man saying “ah” (the vowel), woman saying “la” (the consonant-vowel), baby cooing, woman singing) and distractors—environmental non-human sounds (paper being crumpled, gunshot, door slamming, dog barking, police siren, gong sounding, helicopter, doorbell, glass breaking, food frying, zip being closed). Both sets are part of a validated battery of environmental sounds [16]. Each sound was 500 ms in duration, including 10 ms linear rise and fall time (Audacity 2.1.0 http://audacity.sourceforge.net/ (accessed on 28 April 2026)). The target sound was presented in a pseudo-randomised fashion 9 times at each of the 9 positions. The task lasted 7 min and 40 s.

In either task, the length of intervals between trials was jittered and lasted 0.2 to 2 s, with a step of 0.2 s. Response time was limited to 2 s. At the end of each trial, the laser returned automatically to the midline position. Performance was assessed in terms of localisation errors, expressed in absolute values (°) and corresponding to the difference between the location indicated by the subject and the actual position of the target sound.

### 2.4. Visuo-Spatial and Attentional Tasks

Three standard tasks from the Test for Attentional Performance battery (TAP [17]) were used to assess sustained attention, inhibition and verbal working memory, respectively:

- The phasic alert task, with or without alerting sound, lasting 4.5 min: response times were analysed.
- The Go/No-go task, which lasted 2.75 min: accuracy and response times were analysed.
- The 2-back task, with visually presented numbers, lasting 5 min: accuracy and response times were analysed.

Visuo-spatial short-term memory performance was assessed with the Corsi Block-Tapping task [18], which lasted approximatively 5 min. Performance was assessed in terms of span, i.e., the number of blocks presented in a sequence, which was successfully reproduced by the subject.

### 2.5. Statistical Analyses

Group differences between professional firefighters and control participants in performance on the three TAP subtests [17] and the Corsi Block-Tapping task [18] were assessed using independent-samples *t*-tests.

Performance in the two sound localisation tasks was analysed using mixed-design analyses of variance (ANOVA), with Task (meaningless single sounds; meaningful sounds with distractors) and Position (−80° to 80°) as within-subject factors, and Group (firefighters, controls) as a between-subject factor. Within-subject factors correspond to repeated measurements within the same participants, whereas the between-subject factor reflects differences between independent groups. This analysis allows assessment of the main effects of each factor as well as their interactions. When significant interactions were observed, post hoc comparisons were conducted to examine group differences at each position separately. To control for multiple comparisons, *p*-values were adjusted using the Holm–Bonferroni method [19]. In addition, for each task analysed separately, two-way mixed-design ANOVAs were conducted with Group as a between-subject factor and Position as a within-subject factor.

## 3. Results

### 3.1. TAP and Corsi Block Tasks

No significant differences were found between professional firefighters and control participants on any of the three subtests of the Attentional Performance battery (TAP) or on the Corsi Block-Tapping task (Table 1).

### 3.2. Auditory Spatial Tasks

**Figure 2:**
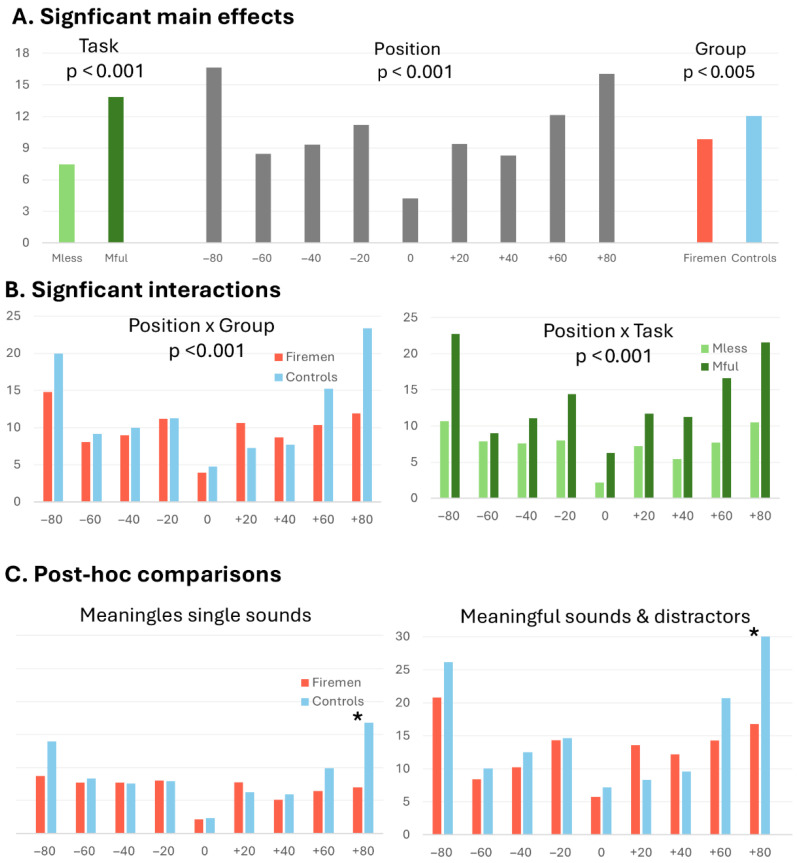
Analysis of localisation errors by means of ANOVA Task × Position × Group. (**A**) The significant main effect Task was driven by smaller errors in the localisation of meaningful single sounds, the main effect of Position by smaller errors at central rather than peripheral positions, and the main effect of Group by smaller errors made by firefighters rather than controls. (**B**) The significant interaction Position × Group was driven by more accurate performance by firefighters than controls at peripheral positions. The significant interaction Task × Position was driven by more accurate performance in locating meaningless single sounds, rather than meaningful sounds with distractors, at peripheral positions. (**C**) Post hoc analysis comparing errors when locating meaningless single sounds (**left**) or meaningful sounds with distractors (**right**). Asterisks mark significant difference in performance between firefighters and controls (*p* < 0.0001; Bonferroni–Holm correction for multiple comparisons).

The 3-way ANOVA with within-subject factors Task (meaningless single sounds; meaningful sounds with distractors) and Position (80°, −60°, −40°, −20°, 0°, 20°, 40°, 60°, 80°) and the between-subject factor Group (Firefighters, Controls) yielded significant main effects: Task (F(1, 61) = 71.61, *p* < 0.001), Position (F(8, 488) = 37.14, *p* < 0.001) and Group (F(1, 61) = 8.42, *p* = 0.005) (Figure 2A). The localisation errors tended to be smaller when localising meaningless single sounds (main effect Task), for items presented at central as compared to peripheral positions (main effect Position), and by firefighters compared to controls (main effect Group).

The interactions Position × Group (F(8, 488) = 9.36, *p* < 0.001) and Task × Position (F(8, 488) = 7.04, *p* < 0.001) were significant (Figure 2B). The former was driven by firefighters performing more accurately at peripheral positions than controls, and the latter by greater differences between peripheral vs. central positions when meaningful sounds with distractors were tested, as compared to meaningless single sounds. The interaction Task × Group (F(1, 61) = 0.04, *p* = 0.838) and the triple interaction Task × Position × Group (F(8, 488) = 0.67, *p* = 0.64) were not significant.

### 3.3. Explicit Localisation of Meaningless, Single Sounds in a Dark Environment

The precision in localising sounds in this condition was assessed by means of localisation errors, expressed in absolute numbers, and analysed with a 2-way ANOVA with Group (professional firefighters, control subjects) as between-subject factor and position (80°, −60°, −40°, −20°, 0°, 20°, 40°, 60°, 80°) as within-subject factor. The interaction Group × Position (F(8, 496) = 5.42, *p* < 0.001) as well as main effects of Group (F(1, 62) = 8.19, *p* = 0.006) and of Position (F(8, 496) = 27.81, *p* < 0.001) were significant. Post hoc analysis compared the performance between groups for each position separately. Professional firefighters localised meaningless single sounds significantly more accurately than control subjects at the right-most (80°) position (*p* < 0.0001; Bonferroni–Holm correction for multiple comparisons) (Figure 2C).

### 3.4. Explicit Localisation of Meaningful Target Sounds in a Dark Environment, While Two Simultaneous Distractors Are Presented at Other Positions

The precision in localising sounds in this condition was assessed by means of localisation errors, expressed in absolute numbers, and analysed with a 2-way ANOVA with Group (professional firefighters, control subjects) as between-subject factor and position (80°, −60°, −40°, −20°, 0°, 20°, 40°, 60°, 80°) as a within-subject factor. The interaction Group × Position (F(8, 488) = 5.63, *p* < 0.001) and the main effect of Positions (F(8, 488) = 13.36, *p* < 0.001) were significant. The main effect of the factor Group was not significant (F(1, 61) = 2.90, *p* = 0.94). Post hoc analysis compared the performance between groups for each position separately. Professional firefighters localised meaningless single sounds significantly more accurately than control subjects at the right-most (80°) position (*p* < 0.0001; Bonferroni–Holm correction for multiple comparisons) (Figure 2C).

## 4. Discussion

### 4.1. Firefighters’ Expertise in Locating Sounds

We demonstrate here that professional firefighters perform better than controls at indicating the position of single meaningless sounds. They are also more proficient at localising meaningful sounds, which are presented with simultaneous distractors. Their advantage at locating sounds could not be ascribed to an attentional bias; firefighters and controls did not differ in their performance in a series of visuo-spatial and attentional tasks, including phasic alertness, Go/No-go and 2-back tasks, as well as the visuo-spatial short-term memory span.

### 4.2. Auditory Spatial Expertise in Professional Groups

Only a few studies investigated auditory spatial expertise in professional groups. Orchestra conductors, as compared to professional piano players and non-musicians, performed better at target detection within the right peripheral space [20]. The targets were pink noise bursts, presented infrequently among pink noise bursts of another bandwidth. Event-related potentials recorded during the task revealed in conductors, but not in the other two groups, attentional spatial gradients for peripheral stimuli. Thus, conductors benefit from behavioural selectivity for sound sources located in the peripheral auditory space [21]. It is interesting to note that professional firefighters, who have a very different professional exposure to sound than orchestra conductors, nevertheless also have better sound localisation performance in the right peripheral space.

Higher sensitivity to binaural cues, which are used in sound localisation, to interaural level differences and to interaural time differences has been reported in musicians. The advantage in spatial hearing tasks was only presented when the musicians were highly trained, had started their training early in life and continued to play. In this particular context, the advantage was larger in male than female musicians [22]. The advantage of male as compared to female highly trained musicians does not reflect a predisposition of the general population. The discrimination threshold of small changes in interaural level differences was found to be lower for male than female participants and no difference was found when interaural time differences were used [22].

Soldiers and marksmen with extensive experience with small firearms were asked to distinguish the direction of fire. Two conditions were used in an EEG oddball paradigm. Fire from small arms was presented as forward fire, i.e., as if aimed at the subject, or as off-axis fire, not aimed at the subject. The former constituted the infrequent, oddball stimuli, the latter the standard stimuli. The oddball vs. standard stimuli yielded different activations within Brodmann’s area 19 in the right hemisphere [23]. In a second study, soldiers and marksmen were compared with non-experts; although they did not perform better or faster, they differed in respect to activation patterns [24]. Although this task involves spatial judgement about the direction of fire—forward or off-axis—the discrimination between the two conditions does not rely on auditory spatial cues, but on the detection of a specific sound pattern. The ballistic crack, a brief sharp sound produced as the bullet breaks the sound barrier, is heard only when fire is aimed at the subject.

### 4.3. Dual Contribution of Auditory Spatial Cues

Auditory spatial cues contribute to two distinct aptitudes, which rely at the cortical level on two distinct, partially segregated networks [4]. One of the aptitudes involves explicit localisation of sounds by word or by deed [5,6]. Imaging studies have shown that explicit sound localisation relies on a predominantly right-hemispheric parieto-frontal network, often referred to as the auditory Where stream, which is partially distinct from the bilateral temporo-frontal network involved in sound recognition, referred to as the auditory What stream [25,26,27,28,29,30,31,32,33,34,35,36,37,38]. The functional independence of the auditory What and Where networks was demonstrated in lesion studies by the double dissociation between deficits in sound localisation vs. sound recognition [39,40,41,42,43,44].

The other aptitude involves the use of auditory spatial cues for the segregation of simultaneously occurring sound sources, the tracking of individual sound sources across space and the orienting of attention towards a specific sound source. This contribution of auditory spatial cues can occur without conscious awareness and is referred to as implicit [45,46]. It relies on encoding, which combines sound meaning and sound location [4,47]. This combined representation of location and meaning is present in specific early-stage auditory areas [48] and in fronto-temporal regions [49,50,51], predominantly within the left hemisphere. As demonstrated in numerous studies [32,48,49,50,51,52,53,54,55,56], the right parietal cortex is not involved in the implicit representation, in striking contrast to its involvement in explicit sound localisation [57]. Explicit sound localisation and the implicit use of auditory spatial cues are functionally independent, as demonstrated in lesion studies by double dissociation of the corresponding deficits [58] and differences in site lesions [46].

Professional firefighters performed significantly better than controls when using auditory spatial cues for explicit sound localisation. Further studies are needed to determine whether the representation of spatial positions within the fronto-parietal Where auditory network differs between firefighters and controls.

Professional firefighters also excelled at a task which required explicit and implicit use of auditory spatial cues. When localising meaningful sounds, here sounds produced by humans, they needed to distinguish them from two simultaneously occurring distractors, here non-human environmental sounds. For this, they may rely on location-linked representation of sound objects, which is distinct from the auditory Where network [4,47]. Future investigations need to determine how firefighters perform at segregating simultaneous sound sources on the basis of spatial cues. Little is known currently about the plasticity of the location-linked representation of sound objects and its contribution to explicit sound localisation. Pioneering studies have demonstrated repetition suppression effects: when the same object was presented several times at the same position, the relevant neural activity decreased. This decrease did not occur when the object changed position between repetitions [48,53,54]. Further studies should address the effects of short-term and long-term training and the interplay between the implicit and explicit use of auditory spatial cues.

## 5. Conclusions

When localising meaningless single sounds, firefighters performed significantly better than control subjects at peripheral positions, in particular on the right side. Their advantage is reminiscent of the performance of orchestra conductors, who were reported to perform better at target detection within the right peripheral space [20]. Firefighters also perform better at localising meaningful sounds among simultaneous distractors. The proficiency of firefighters to localise sounds needs to be taken into consideration when planning and carrying out interventions.

## Figures and Tables

**Table 1:** Performance of professional firefighters and control subjects in subtests of the Attentional Performance battery [17] as well as the Corsi Block-Tapping task [18].

Task	Scores		Statistical Comparison (*p*)
	Professional Firefighters	Control Subjects	
**Alertness task, TAP battery**			
Response time without sound alert [ms]	230 ± 30	240 ± 38	0.296
Response time with sound alert [ms]	232 ± 37	238 ± 36	0.493
**Inhibition Go/No-Go task, TAP battery**			
Response time [ms]	398 ± 61	394 ± 60	0.801
Accuracy [%]	100% ± 10%	99% ± 6%	0.307
**Verbal working memory 2-back task, TAP battery**			
Response time [ms]	691 ± 178	702 ± 121	0.784
Accuracy [%]	80% ± 20%	89% ± 14%	0.055
**Spatial working memory Corsi Block-Tapping task**			
Span	6.3 ± 1.4	6.8 ± 1.2	0.237

## Data Availability

The raw data supporting the conclusions of this article is available upon request to the corresponding author.
